# Shorter Leukocyte Telomere Length in Midlife Women with Poor Sleep Quality

**DOI:** 10.4061/2011/721390

**Published:** 2011-10-20

**Authors:** Aric A. Prather, Eli Puterman, Jue Lin, Aoife O'Donovan, Jeffrey Krauss, A. Janet Tomiyama, Elissa S. Epel, Elizabeth H. Blackburn

**Affiliations:** ^1^Robert Wood Johnson Foundation Health and Society Scholars Program, Center for Health and Community, University of California San Francisco, 3333 California Street, Suite 465, San Francisco, CA 94118, USA; ^2^Department of Psychiatry, University of California, San Francisco, CA 94143, USA; ^3^Department of Biochemistry, University of California, San Francisco, CA 94143, USA; ^4^Department of Psychology, Rutgers University, New Brunswick, NJ 08901, USA; ^5^Department of Nutrition, Rutgers University, New Brunswick, NJ 08901, USA

## Abstract

*Background*. Accumulating evidence supports leukocyte telomere length (LTL) as a biological marker of cellular aging. Poor sleep is a risk factor for age-related disease; however, the extent to which sleep accounts for variation in LTL is unknown. *Methods*. The present study examined associations of self-reported sleep duration, onset latency, and subjective quality with LTL in a community-dwelling sample of 245 healthy women in midlife (aged 49–66 years). *Results*. While sleep duration and onset latency were unrelated to LTL, women reporting poorer sleep quality displayed shorter LTL (*r* = 0.14, *P* = 0.03), independent of age, BMI, race, and income (*b* = 55.48, SE = 27.43, *P* = 0.04). When analyses were restricted to participants for whom sleep patterns were chronic, poorer sleep quality predicted shorter LTL independent of covariates and perceived psychological stress. *Conclusions*. This study provides the first evidence that poor sleep quality explains significant variation in LTL, a marker of cellular aging.

## 1. Introduction

The elderly (65 year or older) population is growing at a remarkable rate, expected to exceed 72 million in the United States by 2030 [1]. This growth is likely to lead to increasing prevalence in age-related disease, including cardiovascular disease, diabetes, and various forms of cancer. The burden of this population on the health care system will be formidable, thus, highlighting the importance of identifying markers of cellular aging implicated in the onset and progression of disease. Emerging evidence supports telomere length as a correlate and potential mechanism underling rates of diseases. Telomeres are DNA-protein complexes that cap chromosomal ends, conferring chromosomal stability [2, 3]. Shorter leukocyte telomere length is a putative risk factor for several chronic conditions, including hypertension [4], atherosclerosis [5], type 2 diabetes mellitus [6, 7], and predicts risk for cardiovascular and all-cause mortality [8–10]. 

There are several psychological and behavioral factors associated with telomere length, including psychological distress [11], personality characteristics [12], poor diet [13], cigarette smoking [14, 15], and leading a sedentary lifestyle [16, 17]. Interestingly, sleep, a modifiable health behavior that often worsens with age [18] and is repeatedly predictive of rates of chronic disease [19–21], has yet to be evaluated. Prior laboratory and epidemiologic evidence supports associations between disrupted sleep and several plausible biological pathways to disease, including alterations in cellular immune function [22]; however, this work has not extended to markers of cellular aging, such as telomere length. 

The aim of the current study was to examine the associations of sleep duration and sleep quality with leukocyte telomere length (LTL) in a sample of healthy women in midlife. Based on the existing sleep literature, it was hypothesized that shorter sleep duration and poorer sleep quality would be related to shorter LTL. 

## 2. Methods

### 2.1. Participants

Two hundred and sixty-three healthy women between the ages of 50 and 65 were recruited through flyers and posters in the San Francisco Bay Area. Participants are part of an ongoing prospective study on telomere length change over the course of a year, examining the effects of knowing one's telomere length on behavior and psychological stress. Participants were excluded from the study if they had (a) any cancer diagnosis within the past 5 years, (b) any cancer treatment, including chemotherapy, radiation, and/or long-term immunosuppressant therapy, within the past 10 years, (c) diagnosis of autoimmune disorder (e.g., rheumatoid arthritis), or (d) current smoker status. Fifteen participants with lifetime histories of cancer were further excluded from this study. Participants were free of serious illness as assessed by self-report at time of participation. In the current study, two additional participants had missing LTL, and one was missing subjective sleep quality, leaving 245 participants with complete data. The study protocol was approved by the Institutional Review Board of the University of California, San Francisco.

### 2.2. Procedure

Interested women who met eligibility criteria by telephone screening provided written, informed consent upon arrival at the first visit. All women participated in a morning fasting blood draw for LTL measurement. Trained research assistants then completed anthropometric measurements on all women in a separate room. Finally, they completed a battery of sociodemographic, psychological, and health behavior questionnaires immediately after eating a provided breakfast.

### 2.3. Sleep Measures

Measures of self-reported sleep were obtained using questions adapted from the Pittsburgh Sleep Quality Index [23]. Specifically, participants were asked to respond to the following regarding their sleep in the past week: “When have you usually gone to bed at night?”; “How long in minutes has it usually taken to fall asleep each night?”; “When have you usually gotten up in the morning?”; “How many hours of actual sleep did you get per night?”; and “How would you rate your sleep quality overall?” ((1) very bad to (5) very good), thereby, providing self-report measures of time in bed, duration, sleep onset latency, and sleep quality, respectively. These single-item sleep measures are common in epidemiologic studies and have been linked to a variety of negative health outcomes [24–27]. Finally, participants were asked if their sleep patterns over the past week represent their typical sleep pattern over the past 3 months (typical versus atypical). This provided important information regarding the chronicity of the reported sleep measures.

### 2.4. Psychological Measures

Participants also completed the Perceived Stress Scale [28] to assess levels of psychological stress. This 10-item measure is widely used for assessing stress perceptions, including ratings of feeling overwhelmed, out of control, and stressed over the past month.

### 2.5. Leukocyte Telomere Length

Fasting morning blood was collected in 10-mL heparin tubes (Becton-Dickinson, Franklin Lakes, NJ, USA). Whole blood was stored in 1 mL aliquots in screw-cap eppendorf tubes at −80°C, and DNA was prepared in batches using QIAamp blood mini kit (QIAGEN Inc.). Telomere length was measured by qPCR adapted from the published methods by Cawthon [29] and Lin et al. [30]. Carried out on a Roche Lightcycler 480 real-time PCR machine with 384-tube capacity (Roche Diagnostics Corporation, Indianapolis, IN), the telomere thermal cycling profile consists of cycling for T(telomeric DNA) PCR: denature at 96°C for 1 second, anneal/extend at 54°C for 60 seconds, with fluorescence data collection, 30 cycles. Cycling for S (single copy gene) PCR: denature at 95°C for 15 seconds, anneal at 58°C for 1 second, extend at 72°C for 20 seconds, 8 cycles, followed by denaturing at 96°C for 1 second, annealing at 58°C for 1 second, extending at 72°C for 20 seconds, holding at 83°C for 5 seconds with data collection, 35 cycles. The primers for the telomere PCR are tel1b (5′-CGGTTT(GTTTGG)5GTT-3′), used at a final concentration of 100 nM, and tel2b (5′GGCTTG(CCTTAC)5CCT-3′) used at a final concentration of 900 nM. The primers for the single-copy gene (human beta-globin) PCR are hbg1 (5′GCTTCTGACACAACTGTGTTCACTAGC-3′), used at a final concentration of 300 nM, and hbg2 (5′-CACCAACTTCATCCACGTTCACC-3′), used at a final concentration of 700 nM. The final reaction mix contained 20 nM Tris-HCL, pH 8.4: 50 mM KCl; 200 *μ*M each dNTP; 1% DMSO; 0.4x Syber Green (Invitrogen, Carlsbad, Calif, USA); 22 ng E. coli DNA (MP Biomedicals, Solon, Ohio, USA); 0.4 Units of Platinum Taq DNA polymerase (Invitrogen, Carlsbad, Calif, USA) and 0.5–10 ng of genomic DNA per 11 *μ*L reaction. Tubes containing 26, 8.75, 2.9, 0.97, 0.324, and 0.108 ng of a reference DNA (from HeLa cancer cells) were included in each PCR run so that the quantity of the targeted templates in each sample could be determined relative to the reference DNA sample by the standard curve. Additional details of the method can be found in Lin et al. [30]. Telomere length is presented in terms of base pairs. This method of LTL measurement is reliable and comparable to alternative methods, such as southern blot [31].

### 2.6. Statistical Analyses

All analyses were performed using SPSS version 18.0. Linear regression models examined associations of sleep variables with LTL, adjusting for a set of standard covariates known to be associated with LTL in past studies (chronological age, body mass index, race, and income). To determine if the relationship between sleep and LTL was better accounted for by psychological stress, separate analyses were conducted, entering the set of standard covariates in the first step, perceived stress in the second step, and the sleep variable in the third step. Finally, because prior evidence suggests that persistent psychosocial factors have a greater influence on telomere length than more transient factors [11], a final analysis was conducted whereby we restricted the sample to only those participants for whom the sleep measures obtained represented their “typical” sleep. Importantly, because previous investigations support a U-shaped association of sleep duration with mortality [32], nonlinear models were also examined. 

## 3. Results

### 3.1. Descriptive Statistics

Means and standard deviations for the study sample are presented in Table 1. Correlational analyses revealed an inverse association between LTL and BMI (*r* = −0.17, *P* < 0.01). LTL was unrelated to age and race, as expected given the restricted age range and homogeneity of the sample's ethnicity/race (84.5% Caucasian). LTL was also unrelated to income, depressive symptoms, and levels of perceived stress. Older participants were more likely to report poorer sleep quality (*r* = 0.21, *P* = 0.001) and longer sleep onset latency (*r* = −0.15, *P* = 0.02). As expected, participants reporting shorter sleep duration, poorer sleep quality, and longer sleep onset latencies were more likely to report higher levels of perceived stress (*P'*s < 0.05).

### 3.2. Sleep and Telomere Length

Bivariate correlations between LTL and measures of time in bed sleep (*r* = −0.06, *P* = 0.36), duration (*r* = 0.01, *P* = 0.92), and sleep onset latency (*r* = −0.11, *P* = 0.09) failed to support the hypothesis that these variables would be associated with LTL. Moreover, there was little support for a non-linear association between LTL and sleep duration. However, poorer subjective sleep quality was associated with shorter LTL (*r* = 0.14, *P* = 0.03). Table 2 displays the results of linear regression models adjusting for study covariates (age, BMI, race, and income). In this regard, poor sleep quality predicted shorter LTL independent of covariates. 

**Figure 1:**
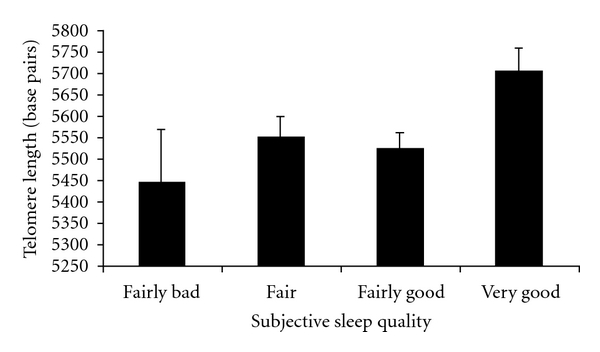
Subjective sleep quality is associated with leukocyte telomere length among participants for whom sleep quality rating reflected their “typical” sleep (*n* = 201), adjusting for age, race, BMI, income, and perceived stress.

In order to determine if the relationship between sleep quality and LTL was better accounted for by levels of perceived stress, we computed a separate regression. In this regard, adjusting for levels of perceived stress, the relationship between sleep quality and LTL was reduced below statistical significance (*b* = 47.36, SE = 29.52, *P* = 0.11). In a follow-up analysis, we examined if the same relationship held among participants for whom self-reported sleep quality was typical for the past 3 months (*n* = 201). As displayed in Figure 1, when the sample is restricted to these participants, sleep quality emerges as an independent predictor of LTL, which remains significant after adjustment for covariates and perceived stress (*b* = 68.45, SE = 34.44, *P* = 0.05). 

## 4. Discussion

This study provides initial evidence for an association between sleep quality and leukocyte telomere length, a marker of immune cell aging, among a sample of healthy midlife women. Specifically, we found that women reporting poorer subjective sleep quality had shorter LTL, independent of age, body mass index, race, and income, thus, providing preliminary evidence that LTL may reflect a potential biological mechanism linking sleep and age-related disease. 

In the full sample (*N* = 245), levels of perceived stress accounted for variance in the relationship between sleep quality and telomere length. However, when analyses were limited to those participants whose sleep quality in the past week was typical of their sleep over the past three months (*N* = 201), sleep quality was a significant predictor of LTL, independent of perceived stress, indicating that the chronicity of sleep problems may be an important qualifier in this association. This is consistent with a prior investigation of caregiving stress and LTL, which found that duration of caregiving and not merely being a caregiver was associated with telomere attrition [11]. 

The biological mechanisms underlying the association between sleep quality and telomere attribution remain to be elucidated. Indeed, disrupted sleep quality has been associated with cortisol secretion [33], enhanced autonomic activation [34], and elevated proinflammatory cytokine production [35], which may contribute to variation in LTL. Recent work suggests that LTL, in addition to being a promising biomarker of cellular aging, may also be an important contributor to disease pathogenesis [36]; rodent models lacking telomerase have implicated telomere attrition as a mechanism leading to mitochondrial damage, increased oxidative stress, and structural and functional damage to cardiac tissue [36]. Relatedly, it is possible that chronically disturbed sleep is related to diminished telomerase activity; however, no study has yet examined whether telomerase activity varies by quality or duration of sleep.

 There are a number of limitations of the current study. The cross-sectional nature of the study precludes any causal inferences regarding sleep quality and LTL. In addition, we relied on self-reported sleep measures, and more than 67% of the participants described their sleep as “fairly good” or “very good.” Future studies would benefit from using more ecologically valid measures of sleep, including in-home polysomnography and actigraphy, as well as sampling within a population with greater variation in quality of sleep; the latter could be accomplished by including a more comprehensive measure of subjective sleep quality. Importantly, participants in the current study were predominantly Caucasian, well educated, in a high-income bracket, and included only women. Thus, the present findings are potentially restricted to this sample of women, and the possibility exists that other components of sleep, such as duration and latency, would be associated with LTL in a more diverse sample of participants. Furthermore, while perceived stress was assessed, other related psychological constructs and measures of stressful life events often related to sleep quality were not tested here. This is important given converging evidence that suggests that childhood trauma is a strong predictor of short LTL in later life [37, 38]. Additional studies are needed to determine the extent to which psychological factors account for the observed association between sleep quality and LTL. Finally, we did not assess for the presence of clinical sleep disorders, such as obstructive sleep apnea (OSA), in this study. This is an important issue given that adults with OSA have been shown to exhibit shorter LTL compared to those without OSA [39]. Notably, the presence of OSA in children is associated with longer LTL [40]. While the influence of OSA on LTL requires further clarity, the association of sleep quality and LTL in the present study was independent of BMI, a common correlate of OSA. 

The alarming increase in the prevalence of age-related diseases suggests that greater attention be placed on identifying biomarkers of cellular aging and modifiable behaviors that may slow the rates of illness. The present study provides the first evidence that poorer subjective sleep quality is associated with shorter LTL, and additional research using prospective samples and objective measures of sleep is warranted. Sleep is a modifiable health behavior, and while the clinical significance of LTL remains to be elucidated, this study provides promising results that sleep quality may be an important factor explaining variation in cellular aging and later health.

## Figures and Tables

**Table 1:** Characteristics of the study sample (*n* = 245 unless otherwise noted).

Variable	Mean or %	SD
Age (years)	57.5	4.4
Race (% Caucasian)	84.5%	
Body mass index (kg/m^2^)	24.4	4.7
Household income (% ≥75 K/year)^a^	67.2%	
Perceived stress (PSS score)^b^	23.2	6.0
Time in bed (hours)^c^	8.0	1.0
Sleep duration (hours)^d^	7.2	1.0
Sleep onset latency (minutes)	17.2	18.9
Sleep quality (1 = very bad, 5 = very good)	3.8	0.8
Telomere Length (T/S converted to base pairs)	5547.0	330.7

^a^ *n* = 235, ^b^ *n* = 241, ^c^ *n* = 238, ^d^ *n* = 243.

**Table 2:** Linear regression models displaying associations of sleep variables with telomere length (base pairs) adjusting for study covariates.

Model	B	SE B	*β*	*t*	*P* value
Time in bed					
Age	−4.29	5.08	−.056	−.845	.399
Race	−67.05	60.30	−.073	−1.11	.267
BMI	−11.43	4.72	−.162	−2.42	.016
Income	18.77	47.11	.026	.398	.691
Time in bed	−20.62	21.56	−.063	−.956	.340

Sleep duration					
Age	−4.09	5.03	−.053	−.798	.426
Race	−47.06	60.02	−.052	−.784	.434
BMI	−11.54	4.67	−.163	−2.47	.014
Income	11.72	46.57	.017	.252	.801
Sleep duration	−2.44	22.26	−.007	−.110	.913

Sleep onset latency					
Age	−5.03	4.99	−.066	−1.01	.315
Race	−53.97	59.32	−.059	−.910	.364
BMI	−11.45	4.62	−.162	−2.48	.014
Income	10.06	46.08	.014	.218	.827
Sleep onset latency	−100.34	71.08	−.092	−1.41	.159

Sleep quality					
Age	−6.04	5.02	−.080	−1.20	.230
Race	−51.03	58.92	−.056	−.866	.387
BMI	−11.19	4.60	−.158	−2.43	.016
Income	2.55	46.07	.004	.055	.956
Sleep quality	55.48	27.43	.133	2.02	.044
